# Associations of instrumental activities of daily living, falls, and depression with subjective cognitive decline

**DOI:** 10.1093/geroni/igaf122.3546

**Published:** 2025-12-31

**Authors:** Idorenyin Udoh, Matthew Smith

**Affiliations:** University of Michigan Flint, Flint, Michigan, United States; Texas A&M University, College Station, Texas, United States

## Abstract

**Background:**

Subjective cognitive decline (SCD) has been associated with challenges performing instrumental activities of daily living (IADL), such as managing finances, doing laundry, or taking medication. Such IADL impairments can contribute to an increased risk of falls and a higher likelihood of depression among older adults. The purposes of this study were to assess the prevalence of SCD among older adults and examine the factors associated with SCD (with an emphasis on IADL difficulties, depression, and falls).

**Methods:**

Data were analyzed from 1,791 older adults ages 65 years and older from the 2022 National Health and Aging Trends Study. A binary logistic regression model was fit to identify the association of sociodemographics, IADL, self-reported falls, and depression on SCD.

**Results:**

About thirteen percent of participants reported SCD, 28.3% reported one or more IADL difficulty, and 39.4% reported depressive symptoms. About 24% reported one or more falls in the past year. Relative to adults ages 65-69 years, those ages 80-84 (aOR=1.74, P = 0.037) and 85-89 (aOR=2.35, P = 0.004) were more likely to report SCD. Participants who reported IADL difficulties (aOR=1.94, P < 0.001), depressive symptoms (aOR=3.33, P < 0.001), and falling in the past year (aOR=1.47, P = 0.017) were more likely to report SCD, respectively.

**Conclusion:**

Findings reinforce the role of functional decline, depression, and falling on cognition among older adults. The interrelated nature of these variables highlights the importance of timely cognitive function screening when subjective memory problems are recognized. Mitigation of risk factors through mental health intervention and fall prevention programming may help offset cognitive declines.

